# Preparation and applications of guard cell protoplasts from the leaf epidermis of *Solanum lycopersicum*

**DOI:** 10.1186/s13007-018-0294-7

**Published:** 2018-03-24

**Authors:** Xuehui Yao, Wenchao Zhao, Rui Yang, Jianli Wang, Fukuan Zhao, Shaohui Wang

**Affiliations:** 1 College of Plant Science and Technology, Beijing University of Agriculture, No. 7 Beinong Road, Changping District, Beijing, 102206 People’s Republic of China; 2 Beijing Key Laboratory of New Technology in Agricultural Application, National Demonstration Center for Experimental Plant Production Education, Beijing University of Agriculture, No. 7 Beinong Road, Changping District, Beijing, 102206 People’s Republic of China; 3 Biological Science and Technology College, Beijing University of Agriculture, No. 7 BeiNong Road, Changping District, Beijing, 102206 People’s Republic of China

**Keywords:** *Solanum lycopersicum*, Guard cell protoplasts, Time saving, Leaf age, Carbonic anhydrases, Hydroponic

## Abstract

**Background:**

Guard cell protoplasts (GCPs) isolated from various plants have proven to be especially useful for studies of signal transduction pathways and plant development. But it is not easy to isolate highly purified preparations of large numbers of GCPs from plants. In this research, our focus is on a method to isolate large numbers of guard cells from tomato leaves. The protocols described yield millions of highly purified, viable GCPs, which are also suitable for studies on guard cell physiology.

**Results:**

We developed an efficient method for isolating GCPs from epidermal fragments of tomato leaves. The protocol requires a two-step digestion to isolate high-quality tomato GCPs. In this procedure, cellulysin (in method L) was replaced by cellulose “Onozuka” RS (in method S) in the first digestion step, which indicated that cellulase RS was more effective than cellulysin. Method S dramatically shortened the time required for obtaining high yields and high-quality GCPs. Moreover, according to the GCP yields, hydroponic plants were more effective than substrate-cultured plants.

**Conclusions:**

In this paper, protocols for large-scale preparation of GCPs and mesophyll cell protoplasts were described, followed by some success examples of their use in biochemical and molecular approaches such as reverse-transcription polymerase chain reaction, real-time polymerase chain reaction and sodium dodecyl sulfate–polyacrylamide gel electrophoresis. The method was proved to be a more efficient GCP-isolating method, capable of providing high yields with better quality in less time.

**Electronic supplementary material:**

The online version of this article (10.1186/s13007-018-0294-7) contains supplementary material, which is available to authorized users.

## Background

Guard cells are specialized leaf epidermal cells that surround natural pores, namely stomata. Changes in guard cell turgor determine the opening or closing, which regulates leaf gas exchange, water transpiration and pathogen defense. Thus, stomata play an important role in the response to external (e.g., light and temperature) and internal stimuli (e.g., endogenous hormones) [1, 2].

The guard cell is a useful model to reveal cells in response to stresses. Guard cell protoplasts (GCPs) isolated from various plants have proven to be especially useful for studies of signal transduction pathways and plant development. Guard cells are an excellent system for studying the transduction of environmental and endogenous signals in plants because guard cells autonomously respond to stimuli [3, 4]. Moreover, guard cells have proven to be an important system for studying secondary messenger regulation of ion channels [5].

The isolation of plant GCPs was first reported in *Allium cepa* and *Nicotiana tabacum* more than 40 years ago [6]. This isolation represented an important step towards the study of cells in isolation. Isolation of GCPs from *Vicia faba*, *Nicotiana glauca*, *Zea mays*, *Commelina communis* and *Beta vulgaris* have also been reported [7–9]. Pandey et al. [10] developed an optimized method to isolate *Arabidopsis* GCPs with high quality and purity. Although GCPs have been isolated from several plant species, care should be taken to minimize harm to protoplasts during the process of cell wall removal [11].

In recent years, improvements in cellulolytic enzymes have made it possible to isolate efficiently GCPs of high quality. In addition, other factors also influence the effectiveness include the digestion temperature, solution pH, presence or absence of bovine serum albumin and the growth status of plants. In addition, it is noteworthy that the optimum osmolality of the isolation enzyme solution must be assessed to avoid swollen GCPs [12].

Tomato has been widely used not only for food but also as a model vegetable that has been used for many investigations involving guard cell physiology [13]. In this study, we developed a time-saving method (Method S) to isolate GCPs from the leaf epidermis of *S. lycopersicum* via mechanical homogenization. Some important modifications were devised based on the traditional GCP preparation (Method L) of *Arabidopsis thaliana* [10] involving guard cell wall digestion by a modified cellulytic enzyme composition, digestion temperature and centrifugation speed for collecting GCPs. Finally, we assessed the viability of protoplasts obtained via these two methods and gave examples of several applications with method S. The modified methods will facilitate the research of plant physiologists, and the time-saving protocol greatly improves the experimental efficiency.

## Methods

### Plant growth

**Fig. 1:**
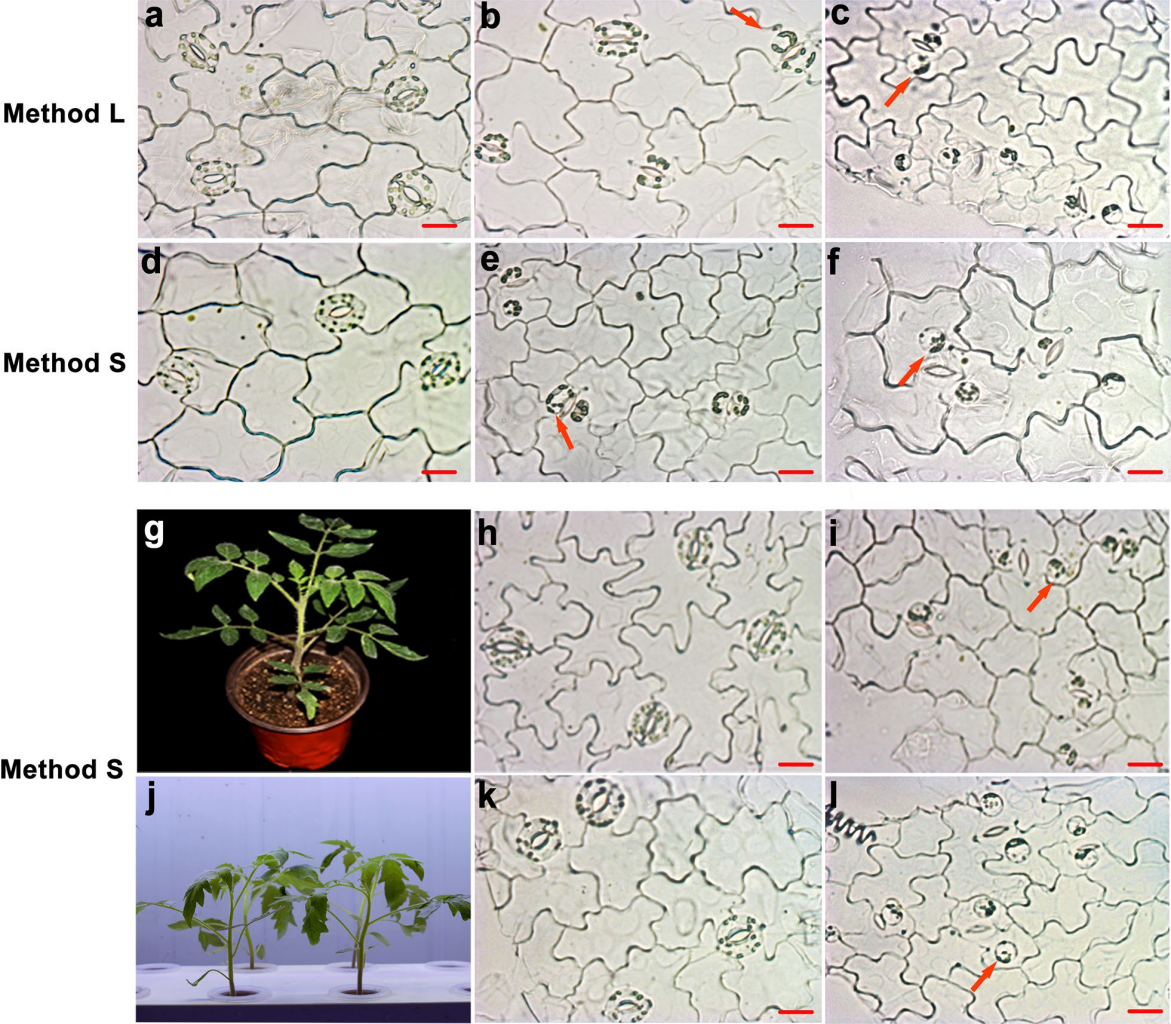
Guard cells at different stages of enzymatic digestion in Method L (**a**–**c**) and Method S (**d**–**l**). **a**–**f** Seven fully expanded leaves of the hydroponic plants selected for GCPs isolation (Additional file 1: Fig. S1). **g**–**i** Seven fully expanded leaves of the substrate culture plants selected for GCPs isolation. **j**–**l** Four fully expanded leaves of the hydroponic plants (younger plants compared with **d**–**f**) selected for GCPs isolation. **a**, **d**, **h**, **k** The status of GCs after 1.5, 0.5, 2 and 0.5 h of digestion in enzyme solution 1. **b**, **e** The status of GCs after 2 and 1 h of digestion in enzyme solution 2. **c**, **f**, **i**, **l** The status of GCs after 3.5, 2.5, 4.5 and 1.5 h of digestion in enzyme solution 2. Scale bars indicate 20 μm

Seeds of tomato CM (Castlemart, wild type) were germinated and grown in a growth chamber under 18 h of light at 27 °C and 6 h of dark at 18 °C in hydroponics or substrate culture. Plants with healthy expanded true leaves were harvested for preparation of GCPs (Additional file 1: Fig. S1 and Fig. 1). The epidermal fragments of true leaves were collected as described for Method L or Method S.

### Preparation of GCPs: Method L

This protocol was developed from methods using *Vicia faba* [14] and *Arabidopsis thaliana* [10] with some modifications involving the enzyme concentration, aperture of the nylon membrane, temperature of digestion, rotation speed and centrifugal speed. The protoplasting solutions are as follows: *Basic medium 1*: 0.5 mM CaCl_2_, 0.5 mM MgCl_2_, 10 μM KH_2_PO_4_, 5 mM 2-[N-morpholino] ethanesulfonic acid hydrate (Mes), and 0.3 M D-mannitol, pH 5.5 (KOH); *Basic medium 2*: 0.5 mM CaCl_2_, 0.5 mM MgCl_2_, 10 μM KH_2_PO_4_, 5 mM 2-[N-morpholino] ethanesulfonic acid hydrate (Mes), and 0.4 M D-mannitol, pH 5.5 (KOH); *Enzyme solution 1*: 1% (w/v) cellulysin^®^ cellulase, *Trichoderma viride*, activity: > 10,000.0 U/g (Calbiochem, USA), 0.1% (w/v) polyvinyl pyrrolidone 40000 (PVP-40), 0.25% (w/v) bovine serum albumin and 0.5 mM L-ascorbic acid, dissolved in basic medium 1; and *Enzyme solution 2*: 1.5% (w/v) cellulose “Onozuka” RS (Yakult Pharmaceutical Industry Co., Ltd, Tokyo, Japan), 0.02% (w/v) Pectolyase Y-23 (Yakult, Japan), 0.25% (w/v) bovine serum albumin and 0.5 mM L-ascorbic acid, dissolved in basic medium 2. It is noteworthy that the enzyme mixture was filtered to remove any insoluble or precipitated materials.

The fully expanded true leaves (weighed ~ 10 g) were harvested from hydroponic tomato plants. Then, the leaves were blended for 1 min (three pulses of 20 s) in a blender (18,000 rpm/min) in 250 ml of cold distilled water. The blended leaf mixture was poured through a 74-μm nylon mesh to remove broken mesophyll cells and rinsed several times with cold distilled water until most of the foam produced by blending had largely disappeared and the epidermal fragments were pale green. Then, the epidermal tissues were transferred into a flask containing 50 ml of enzyme solution 1 that was incubated for 1.5 h at 27 °C in a shaking water bath in darkness, with the shaking speed set to 100 rpm. After 1.5 h of incubation, epidermal fragments were collected on a 58-μm nylon mesh, rinsed gently with basic medium 2 and transferred into 50 ml of enzyme solution 2 in a flask. This solution containing the fragments was then incubated at 25 **°**C in a shaking water bath (70 rpm) until most of the GCPs were released, typically after approximately 3.5 h. To improve the release of GCPs from the epidermal peels, the flask was swirled gently by hand for a few seconds at the end of the digestion. In addition, the peels in the enzyme solution were gently passed several times through a pipettor equipped with a 5-ml tip that was cut to release the GCPs. The enzymolysis effect was assessed under a microscope. The second enzyme digestion mixture was filtered through 37-μm nylon mesh. The epidermal tissues on the mesh were rinsed with basic medium 2 to further release GCPs. The enzyme solution containing the protoplasts was then filtered through a 15-μm nylon mesh, and the released GCPs were collected (in 50-ml centrifuge tubes) by centrifugation for 6 min at 300×*g*. The GCPs were washed three times with basic medium 2 to keep GCPs free from the pellet of mixed cells and other debris. After removing the supernatant, a volume of 5 ml of protoplasts was yielded, which was stored at 4 °C in the dark for the next purification or for other experiments.

### Preparation of GCPs: Method S

This protocol was derived from “Method L”, with one modification involving the composition of enzyme solution 1. Enzyme solution 1 consists of 1.0% (w/v) cellulose “Onozuka” RS, 0.01% (w/v) Pectolyase Y-23, 0.1% (w/v) PVP-40, 0.25% (w/v) bovine serum albumin and 0.5 mM L-ascorbic acid, dissolved in basic medium 1. The improvement in Method S reduced the time required by 2 h compared with Method L. The first enzyme digestion needs only 0.5 h, whereas the second enzyme digestion needs 2.5 h and 1.5 h (with young plants Fig. 1).

### Purification of GCPs

To remove fragments of vascular tissue and contaminants from GCPs, an additional purification step was performed that significantly improves the purity of the GCPs. The purification protocol of the GCPs was according to that of previous methods [10], with modifications adapted for tomato described here. Basic solution 2 was added to the GCPs suspension to give a final volume of 8 ml. Six milliliters of Histopaque (No. 1077, Sigma-Aldrich) was carefully pipetted into the bottom of a centrifuge tube. The GCPs were then carefully layered on top of the Histopaque. The tubes were centrifuged for 20 min at 150×*g*, after which the GCPs were collected from the interface of the two solutions with a Pasteur pipette and transferred to a new test tube. The GCPs were diluted with basic solution 2, and then the tubes were centrifuged for 6 min at 300×*g*. The supernatant was removed, and the isolated GCPs were resuspended in 1 ml of basic solution 2 and kept on ice in the dark until subsequent tests.

### Assessment of protoplast viability

The viability of GCPs was evaluated by fluorescence measurements with fluorescein diacetate (FDA). The dye hydrolysis was observed after mixing GCPs with the incubation mixture for 5 min. The operating concentration of FDA was 0.01% (w/v) dissolved in acetone. Protoplast preparations were viewed and imaged using a Zeiss Axio Observer D1 fluorescence microscope with blue excitation using the FITC filter combination.

### Preparation of MCPs

The method for isolating *Solanum lycopersicum* is modified from that described by Romano et al. [15]. Fully expanded leaves are cut into 0.5 cm^2^ pieces. The leaf pieces are then transferred to 30 ml of enzyme solution: 5 mM Mes, 0.65 M D-mannitol, 1 mM CaCl_2_, 0.5% (w: v) Macerozyme R-10 (Yakult, Japan), 1% (w: v) Cellulase R-10 (Yakult, Japan), 0.25% (w: v) BSA and 0.1% (w: v) PVP-40, pH 5.5 (KOH). Digestion is performed at 25 **°**C for 2 h with slow shaking (40 excursions per min) in a water bath. The enzyme solution turns green after gentle swirling motion, indicating the release of protoplasts. Digestion time depends on the experimental goals and materials used. It is not necessary to release all the protoplasts from leaf pieces. The solution is filtered through 37 μm nylon mesh into a 50-ml centrifuge tube. The leaf pieces retained are rinsed with 20 ml of incubation medium (0.65 M D-mannitol, 1 mM CaCl_2_) and the filtrate is also collected. The released MCPs were collected by centrifugation for 5 min at 100×*g*. The pellet was washed three times with 0.65 M D-mannitol containing 1 mM CaCl_2_. Isolated MCPs were stored on ice in the dark until use.

### Semi-quantitative reverse-transcription PCR analyses

Total RNA was extracted from GCPs and mesophyll cell protoplasts (MCPs) using Arcturus PicoPure RNA Isolation Kit (Applied Biosystems, Cat no. 12204-01, USA) according to the manufacturer’s protocol. Total RNA was reverse-transcribed into cDNA using the Super-Script first-strand synthesis system (TransGen Biotech) according to the manufacturer’s instructions. Semi-quantitative RT-PCR was performed with two genes that were specifically expressed in guard cells or mesophyll cells to assess the protoplast purity. The *Actin* gene (forward primer, 5′-CCTCTCAGTTCCCGTTGAATAG-3′; reverse primer, 5′-TCACCAGAGTCCAACACAA TAC-3′) was used as a control for RT-PCR experiments. The *KAT1* gene (forward primer, 5′-GGAATCAGTTGCCTCCAAGA -3′; reverse primer, 5′-GCTGTGGTCTCCCACATAAA-3′) is an ABA-repressed gene preferentially expressed in guard cells. The *βCAs* gene (*βCA1*, forward primer, 5′- CCTCTTTCTCCCTTAGCTTCATC -3′, reverse primer, 5′-GTGGACCCATCATCA GGAATAG-3′; *βCA2*, forward primer, 5′- CAGT GCTTGTGGAGGTATCAA-3′, reverse primer, 5′- TACGGAAAGAGGAGGAGAAAGA-3′; *βCA3*, forward primer, 5′-TTGTTTCCCTCCAGA ACCTTATC -3′, reverse primer, 5′-GCCT TGATACCTCCACAACTAC-3′) coding for β-carbonic anhydrases plays a direct role in photosynthesis of plants [16].

### Quantitative real-time PCR (qRT-PCR) analyses

The qRT-PCR was performed with four genes that were preferentially expressed in guard cells or depicted as regulators of ABA response and showed transcript level induction or repression in response to MeJA in guard cells. GCPs suspension incubated in 1.5 ml microfuge tubes at room temperature for 10 min, and then treated with 50 μM ± MeJA (final concentration) for 5, 10, 15, 20 min. After the incubation, guard cells were collected simultaneously, frozen and stored at − 80 °C until further analysis. Considering that protoplasting induces the expression of stress-associated genes, two different transcription inhibitors, actinomycin D (33 mg/L) and cordycepin (100 mg/L), were used in all procedures of the isolation, including the first step of blending [1]. The qRT-PCR analyses of early wound-response genes expression with or without the transcription inhibitors were performed.

Total RNA was isolated from the GCPs, using Arcturus PicoPure RNA Isolation Kit (Applied Biosystems, Cat no. 12204-01, USA) according to the manufacturer’s protocol. All RNA samples were digested with RNase-free DNase to remove genomic DNA. The quality and concentration of each of the RNA samples was determined using Namedrop 2000 spectrophotometer (Thermo Scientific). The integrity of RNA was also checked by agarose gel electrophoresis. For real-time PCR, total RNA was reverse-transcribed into cDNA using the Super-Script first-strand synthesis system (TransGen Biotech) according to the manufacturer’s instructions. The cDNA was used as a template to perform real-time PCR with gene-specific primers (Additional file 1: Table S1) and SYBR Green Mix (TaKaRa) in a CFX96 TouchTM Real-Time PCR detection system (Bio-Rad). Actin gene was used as an internal normalization in samples. Fold change in gene expression was calculated using ΔΔCt values.

### Protein extraction and SDS-PAGE analyses

Soluble protein fractions of leaves, MCPs and GCPs were prepared according to Li and Assmann [17]. All subsequent steps were performed at 4 °C. Protoplasts and leaves powder mixed with 40 μl of ice-cold buffer in microfuge tubes. The buffer was 50 mM Tris–HCl, pH 7.5, 1 mM MgCl_2_, 2 mM ethylenediaminetetraacetic acid (EDTA), 0.25 mM ethyleneglycoltetraacetic acid (EGTA), 250 mM sucrose, 2 mM dithiothreitol (DTT), 1 mM phenylmethylsulfonyl fluoride (PMSF) and 10 μg ml^−1^ each of the protease inhibitors leupeptin, pepstatin and aprotinin. The homogenate was kept on ice for 20 min and then centrifuged at 10,000×*g* for 15 min and the supernatant was used for 10% SDS-PAGE. The protein concentrations were measured by the Brandford Kits.

## Results and discussion

### Assessing the enzyme digestion of epidermal peels

Appropriate osmolality contributes to the maintenance of guard cell shape and digestion of the cell wall. The optimum osmolality of isolation buffers should be assessed before preparation of GCPs to ensure that the protoplasts are round rather than prone to rupturing easily. For example, in the isolation of tomato GCPs, as the cell wall was gradually digested in the first-step digestion, the GCPs need higher osmolality during the second-step digestion, which could maintain the integrity of the GCPs. Osmotic responses of GCPs were evaluated in different concentrations of D-mannitol (Additional file 1: Fig. S2). Therefore, we selected 0.3 M D-mannitol solution for the first enzyme digestion and 0.4 M D-mannitol solution for the second enzyme digestion (Additional file 1: Fig. S2).

Various states were observed after digestion by Method L (Fig. 1a–c) and Method S (Fig. 1d–f) with hydroponic plants. Also, the method S was used to isolate the GCPs from the different seedlings status (g-l). Cellulase RS was more effective than cellulysin, since GCPs were becoming spherical at 0.5 h after digestion in method S (Fig. 1d) rather than at 1.5 h in method L (Fig. 1a). In the second digestion, GCPs became round after 1 h of digestion in method S (Fig. 1e) and after 2 h in method L (Fig. 1b). As the incubation time was prolonged, more GCPs were spherical. When most of the GCPs were detached from the epidermal peel in both methods, the enzyme digestion was abolished, and perfectly round GCPs were obtained (Fig. 1c, f). The leaves from younger plants were also collected for testing.

Interestingly, the time taken to extract the GCPs from younger leaves by Method S was shorter than that of the older leaves (Fig. 1f, l). This suggests the time of enzyme digestion may vary depending on leaf age, the younger leaves required less time. Simultaneously, we evaluated the digestion rate of the epidermal peels of substrate-culture plants by these two methods. It showed that Methods S and L took 4 h and 6.5 h, respectively, both of which take more time than the hydroponic-cultivated plants indicating that the growth status of plants greatly impacted on the isolation efficiency (Fig. 1g–l and Additional file 1: Fig. S3a, b).

The general principle of GCP isolation is to release GCPs from epidermal peels through a series of sequential cell wall digestion steps in which epidermal cell walls degraded and GCPs released [10, 14, 18, 19]. In Araujo and Shi’s [20, 21] protocols, it took 2 h or less time to isolate the GCPs of tomato leaves, which was basically consistent with our protocols (Fig. 1j–l). However, protocols, two points were different. First, the two-steps digesting enzymes differ. In their protocol, cellulysin cellulase was used in step one and “Onozuka” RS in step two, whereas the “Onozuka” RS was the only choice in our protocol. Second, the shaking speed was reduced to 100 rpm in our protocol while it is 150 rpm and 120 rpm in Araujo and Shi’s protocols. Due to the result showed that the high shaking speed (150 rpm) is easy to produce sub and incomplete protoplasts (Additional file 1: Fig. S4). Indeed, both enzymolysis efficiency and shaking speed could shorten the time of GCPs isolation. However, based on our data, the efficient enzyme appeared to be a better choice that ensured the intact high-activity GCPs productivity.

### Assessment of purification, viability and yields of GCPs

**Fig. 2:**
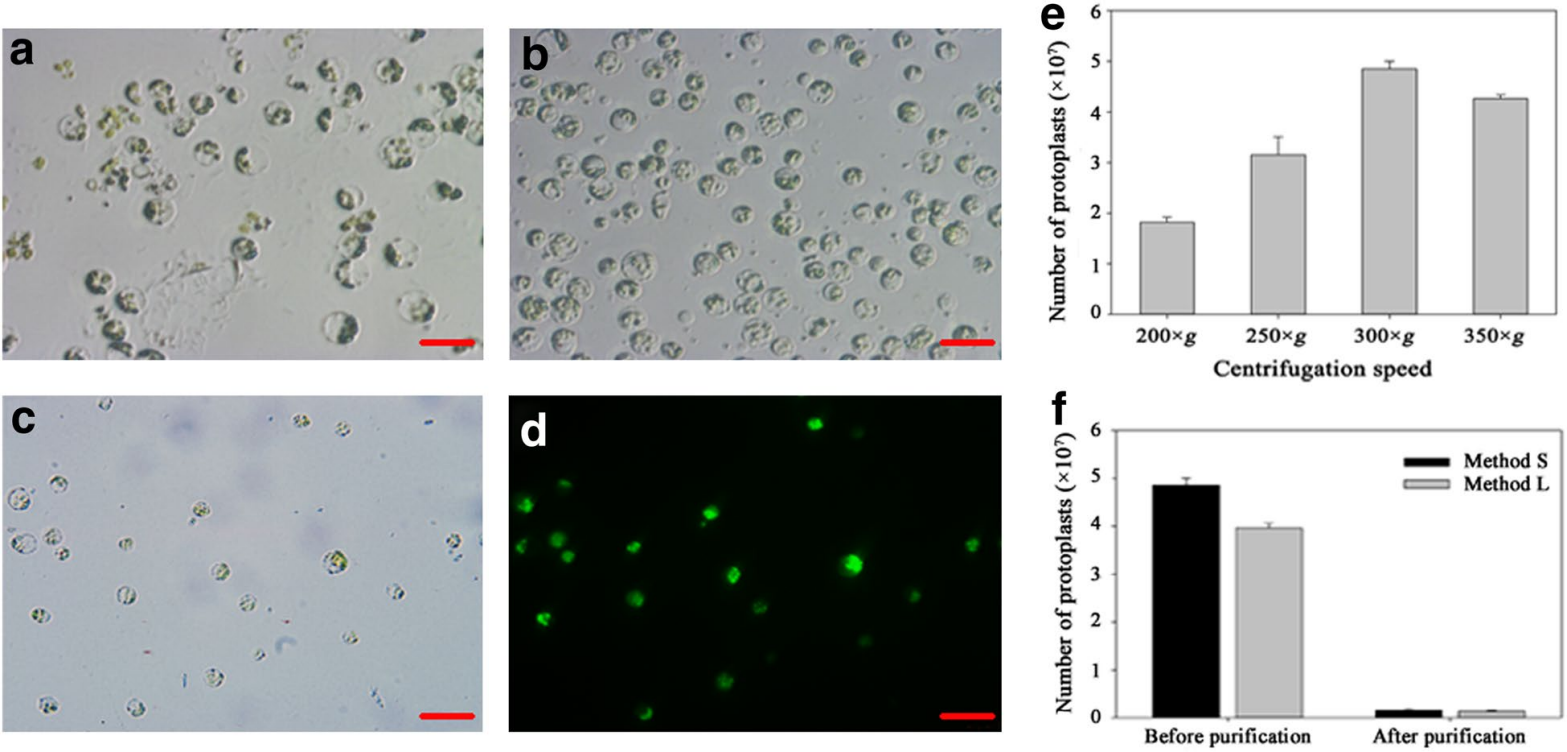
Observation and assessment of GCPs. **a** Mixed GCPs and debris obtained via method S. **b** Status of GCPs after purification via method S. **c**, **d** GCPs stained with fluorescein diacetate (FDA) to assess GCPs viability. Bright-field (**c**) and fluorescence (**d**) micrographs of the preparations are shown from the same field of vision. **e** The yield of GCPs under different centrifugal forces. **f** Comparison of protoplast yields of two methods (L and S) before and after purification. Results are shown as mean (n = 3) ± standard error from different samples. Scale bars indicate 20 μm

Before purification, GCPs and debris were mixed together (Fig. 2a). In this research, four different centrifugal speeds were assessed before purification. The typical yield is 4.85 × 10^7^ GCPs per 10 g of leaves at 300 × g by method S, which is the most suitable centrifugal speed (Fig. 2e). After centrifugation, we obtained high quality of the GCPs that contained intact chloroplasts (Fig. 2b). The guard cells were collected by centrifugation at 300×*g* (before purification) or 150×*g* (purification), resulting in millions of high-quality GCPs. Notably, the protoplast yield decreased at 350×*g*, probably because the high centrifugal speed damaged the protoplasts (Fig. 2e). The centrifugation time also should be as short as possible to minimize damage to the GCPs.

Living cells can be determined by fluorescein diacetate (FDA) staining. The GCPs obtained by both methods were viable in hydroponic culture (Fig. 2d and Additional file 1: Fig. S5d). However, GCPs with high viability were more likely to occur with Method S according to the fluorescence density. It is possible that the longer exposure time in the enzyme solution impacts the physiology of GCPs, which potentially could influence subsequent investigations.

The typical yield was approximately 4–5 × 10^7^ GCPs at a purity 75–80% per 10 g of leaves with the two methods from plants of hydroponic culture before purification. After purification, the yield of the GCPs was approximately 1.2–1.5 × 10^6^ GCPs at a purity 95–98% (calculated on a cell number basis) with these two methods (Fig. 2f). The GCP yields of hydroponic and substrate-cultured plants were compared, and a significant difference was observed. GCP yields of hydroponic plants were much higher than those of substrate-cultured plants before and after purification. In both cultivation patterns, the GCPs yield via Method S was higher than Method L (Fig. 2f and Additional file 1: Fig. S3e). The results showed that culture method influenced the growth status of plants, which significantly impacted the GCPs yield.

### Semi-quantitative reverse-transcription PCR analyses

**Fig. 3:**
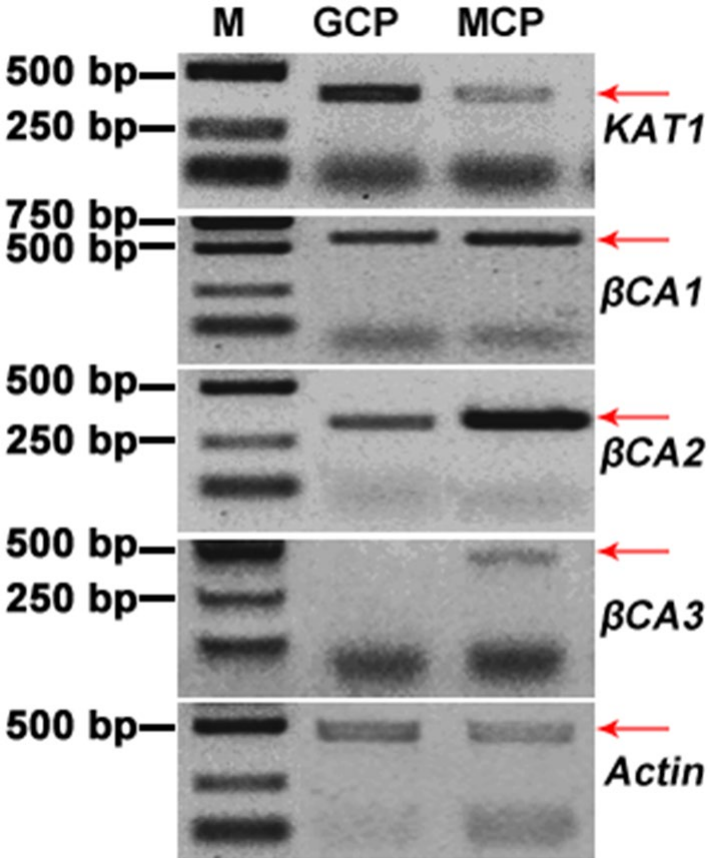
Semi-Quantitative RT-PCR analyses of *KAT1* and *βCA* expression in guard cell (GC) and mesophyll cell (MC) protoplasts. *Actin* gene was used as control. Lane M represents molecular weight markers in bp

We performed semi-quantitative RT-PCR analysis using specific primers for assessing relative gene expression levels in GCPs versus MCPs (The MCPs preparation was shown in Additional file 1: Fig. S6). The *KAT1* gene (as a guard cell-specific gene control) which encodes an inward K^+^ channel showed higher expression in GCPs than MCPs (Fig. 3).

Carbonic anhydrases (CAs) play essential roles in many biological processes especially in photosynthetic organisms [22]. In higher plants, three evolutionarily distinct have been reported namely α-, β-, and γ-CA [23], which independently evolved similar catalytic mechanisms [24, 25]. The β-type CA (βCA) was first discovered in plants in 1990, and there have a moderate number of *βCA* genes [23, 26, 27]. A series of *βCA* genes including *βCA1*, *βCA2* and *βCA3* were identified and characterized in tomato [16]. Phylogenetic analysis based on the amino acid sequences showed *SlCA1*, *SlCA2* and *SlβCA3* had high similarities to *AtβCA2*, especially for *SlβCA3* (Additional file 1: Fig. S7). It showed that three *βCA* genes show significantly lower expression in GCPs than MCPs. Especially, the level of *βCA3* transcripts was hardly detected in GCPs, though the level in MCPs was low either. The members of the *βCA* subfamily in tomato display the same tissue-specific expression patters to the previous research. For example, *AtβCA2* is the most highly expressed *CA* genes in mesophyll cells [28]. Amplification of *actin* transcripts under identical conditions as a control for equal expression in both the cell types and to show amounts of initial cDNA template. Taken together, it proved that Method S could guarantee the purity of GCPs.

### Quantitative real-time PCR analyses

**Fig. 4:**
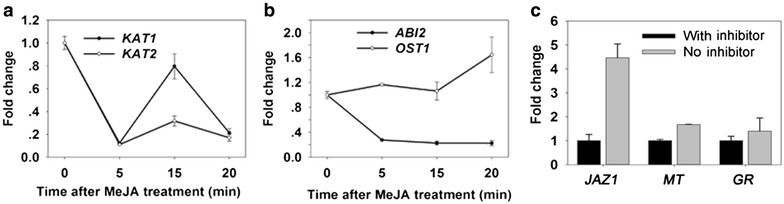
The verification of GCPs in response to external stimuli by quantitative Real-time PCR. **a**, **b** Real-time PCR analyses of ABA-regulated guard cell–expressed genes in guard cells before and after MeJA (50 μM) treatment for various times. **c** Effect of transcription inhibitor (actinomycin D and cordycepin) in wound-responsive gene transcription during GCPs preparation. Results are shown as mean (n = 3) ± standard error

When plants encounter stress conditions and the ABA level rises, then the ABA signaling network that leads to stomatal closure through ABA modulation of ion channel activities, including the regulated efflux of anions and potassium ions and the inhibition of K^+^ import [29, 30]. It has been reported that the activity of KAT1 is inhibited by an elevation of ABA [31]. The core signalosome of ABA signaling including ABA receptors, phosphatases (PP2Cs), and kinases (SnRK2 s) was established [32]. After ABA is perceived by a receptor, the action of PP2Cs such as ABI1 are inhibited and activation of a downstream target of phosphatases-SnRK2 s, such as OST1 [29]. In plants, JA regulates various developmental processes and defense responses. It was well demonstrated that JA is involved in stomatal movement through ABA signaling pathway [29, 33]. In our study, the putative marker genes were induced after JA treatment in guard cells (Fig. 4a, b).

To test whether inhibitors suppressed induction of stress-inducible genes during protoplast isolation, real-time RT-PCR was performed using cDNA synthesized from guard cell RNA from protoplasts prepared in the presence or absence of transcription inhibitors actinomycin D and cordycepin. *JAZ1* gene mRNA levels were used to monitor the effect of the inhibitors because its expression is strongly induced by wounding [34]. Furthermore, besides *JAZ1* gene, we assessed the expression of two other stress-related genes [35]. As shown in Fig. 4, the expression levels of *JAZ1 MT* and *GR* were significantly suppressed by transcription inhibitors (Fig. 4c). The transcription inhibitor could effectively suppressed stress-inducible gene, suggesting that the inhibitor could maintain the intact status from leaves after treatment. It can be used for a large demand of GCPs for instance in omics analysis in tomato.

### SDS-PAGE of soluble proteins

**Fig. 5:**
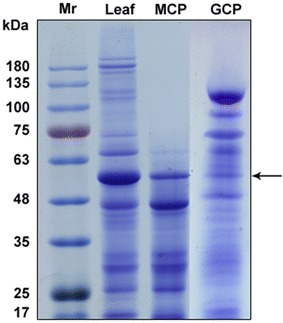
SDS-PAGE analysis of total proteins from *Solanum lycopersicum* leaves, mesophyll cell protoplasts and GCPs. The protein molecular markers (ladder) are in the left lane

Total protein was isolated from leaves, MCPs and GCPs. Twenty-five μg of soluble protein were separated by 10% of SDS-PAGE and stained with Coomassie brilliant blue R250. In the results, leaves, MCPs and GCPs total protein bands were clear. Arrows mark the large (about 55 kDa) subunits of Rubisco (Fig. 5). The method to obtain high resolution protein separation of GCPs provides an excellent way to perform protein tests in guard cells. Interestingly, the high-molecular subunits in MCPs which range in molecular mass from ~ 70 to 180 kDa were hardly detectable compared with GCPs (Fig. 5).

In conclusion, this paper reports that the time-saving (Method S) protocol greatly improves the experimental efficiency. Moreover, the GCPs obtained with Method S had high viability, purity and yield. Therefore, this method provides an alternative pathway for exploring the functional mechanisms of guard cells in tomato, which makes it possible to investigate biological processes and molecular functions in guard cells. Moreover, enzymatic digestion with optimal experimental parameters released large quantities of guard cells, which provides opportunities for omics analyses [36, 37].

## Additional file

**Additional file 1.** **Fig. S1.** Seven fully expanded leaves of the hydroponic plants (older plants) selected for GCPs isolation. **Fig. S2.** Experiments designed to optimize osmolality conditions for isolation. **Fig. S3.** The digestion of the epidermal peels by Method L with substrate-cultured plants. **Fig. S4.** The status of GCPs after 1 h of digestion in enzyme solution 1 with the shaking speed set to 150 rpm. **Fig. S5.** Assessment of purification and viability of GCPs via method L with hydroponic plants. **Fig. S6.** MCPs preparation before (a) and after (b) purification. **Fig. S7.** Phylogenetic tree of CA genes in different plants. **Table S1.** Primers used in the Real-time RT-PCR analyses performed in this study.
