# Supplementary material for: Preparation and applications of guard cell protoplasts from the leaf epidermis of Solanum lycopersicum
Source: Plant Methods. 2018 Mar 24;14:26. doi: 10.1186/s13007-018-0294-7 (PMC5866509; doi:10.1186/s13007-018-0294-7)
Supplement: Supplementary file 1 — Additional file 1. Fig. S1. Seven fully expanded leaves of the hydroponic plants (older plants) selected for GCPs isolation. Fig. S2. Experiments designed to optimize osmolality conditions for isolation. Fig. S3. The digestion of the epidermal peels by Method L with substrate-cultured plants. Fig. S4. The status of GCPs after 1 h of digestion in enzyme solution 1 with the shaking speed set to 150 rpm. Fig. S5. Assessment of purification and viability of GCPs via method L with hydroponic plants. Fig. S6. MCPs preparation before (a) and after (b) purification. Fig. S7. Phylogenetic tree of CA genes in different plants. Table S1. Primers used in the Real-time RT-PCR analyses performed in this study. [file 13007_2018_294_MOESM1_ESM.docx]

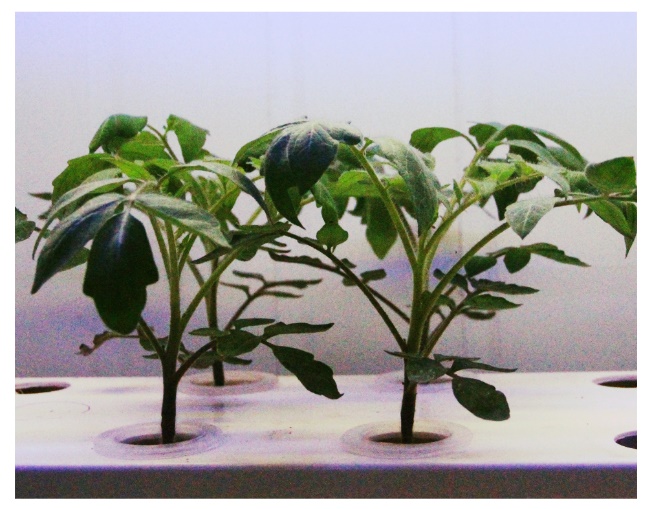
**Additional file**

**Fig. S1.** Seven fully expanded leaves of the hydroponic plants (older plants) selected for GCPs isolation.


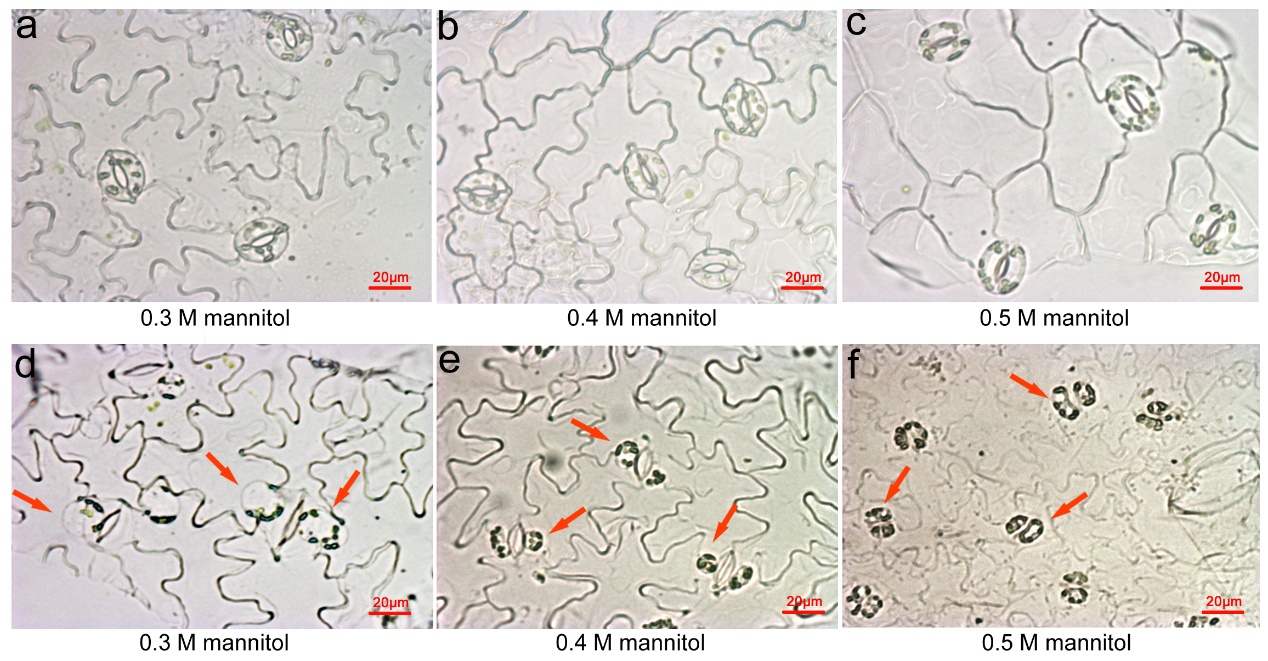


**Fig. S2.** Experiments designed to optimize osmolality conditions for isolation. a-c: The first enzyme solution containing 0.3 M, 0.4 M or 0.5 M mannitol. d-f: The second enzyme solution containing 0.3 M, 0.4 M or 0.5 M mannitol. Plump guard cells were observed after the first cell wall digestion in the low-osmolality (0.3 M) solution (a-c). Round, high-quality protoplasts were released after the second digestion in 0.4 M mannitol solution (e), while the GCPs were swollen or oddly shaped in the 0.3 and 0.5 M solutions (d and f). As the cell wall became partially digested, the osmolality of the second digestion step was usually higher than the first step.


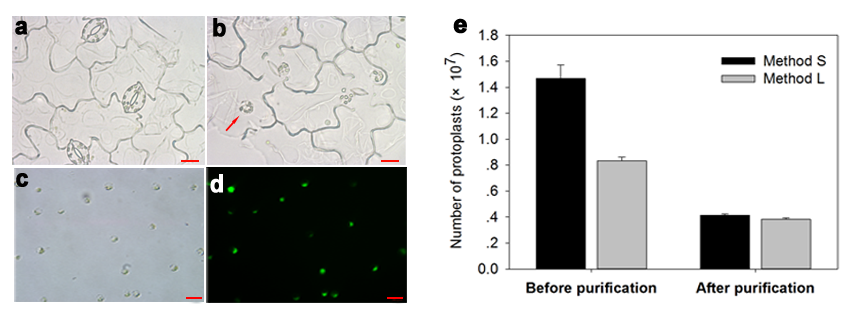


**Fig. S3.** The digestion of the epidermal peels with substrate-cultured plants. a, The status of guard cells after 2 h of digestion in enzyme solution 1. b, The status of GCPs after 4.5 h of digestion in enzyme solution 2. c, d: GCPs stained with fluorescein diacetate (FDA). Bright-field (left) and fluorescence (right) micrographs of the preparations are shown from the same field of vision. e, Comparison of protoplast yields of two methods (L and S) before and after purification. Results are shown as mean (n = 3) ± standard error from different samples. Scale bars indicate 20 μm.


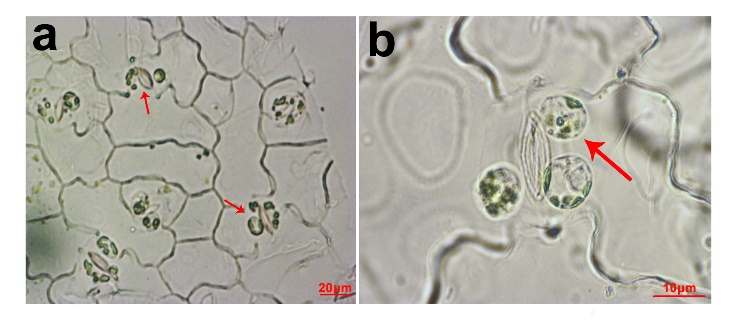


**Fig. S4.** The status of GCPs after 1 h of digestion in enzyme solution 1 with the shaking speed set to 150 rpm. a, b: arrows mark the sub and incomplete protoplasts.

**
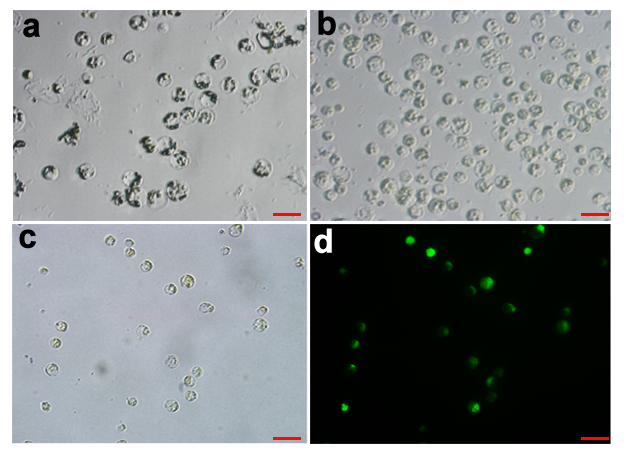
**

**Fig. S5.** Assessment of purification and viability of GCPs via method L with hydroponic plants. a, Mixed GCPs and debris obtained via method L before purification. b, Status of GCPs after purification via method L. c, d: GCPs stained with fluorescein diacetate (FDA) to assess GCPs viability. Scale bars indicate 20 μm.


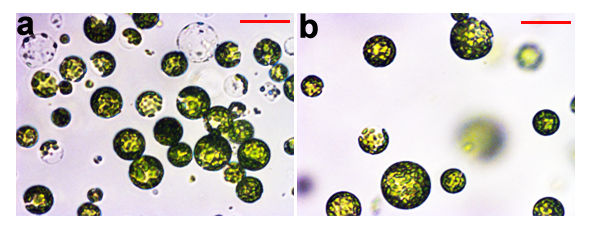


**Fig. S6.** MCPs preparation before (a) and after (b) purification. Scale bars indicate 40 μm.

**
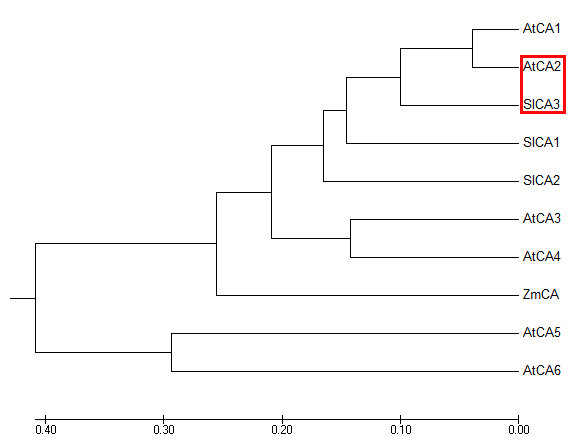
**

**Fig. S7.** Phylogenetic tree of CA genes in different plants.

**Table S1.** Primers used in the Real-time RT-PCR analyses performed in this study.

| Name | sequences |
| --- | --- |
| *Actin-*F | TTGCTGACCGTATGAGCAAG |
| *Actin-*R | GGACAATGGATGGACCAGAC |
| *KAT1-*F | GACCAGATGAGGGAACAAAGAG |
| *KAT1-*R | GCCACTTGAACTGGTACTACAT |
| *KAT2-*F | TCAGCAGCAAACCAACCTGACTC |
| *KAT2-*R | ACTTCTTGGTTCTGTCCGCTTGC |
| *ABI2-*F | AGTGAAACAGACGGGATTGAGC |
| *ABI2-*R | AGCTGTAGATCCAACTGTCTCAGG |
| *OST1*-F | TGGATGGAAGTCCTGCACCAAG |
| *OST1*-R | GTTGTGAATGCAACACCGAGGAC |
| *JAZ1-*F | TTCCCTCAAGGTGGAATGAAGGCT |
| *JAZ1-*R | TCCGAAACTCGGAACCACCAAATC |
| *MT-*F | GCTGTGGATCTAGCTGCAAGTGCG |
| *MT-*R | AAGGGTTGCACTTGCAGTCAGATCC |
| *GR-*F | CGTGCTGTGATACTTGGTGG |
| *GR-*R | TCGTGCAAGGATGCATAGTG |
